# Hepatitis B core VLP-based mis-disordered tau vaccine elicits strong immune response and alleviates cognitive deficits and neuropathology progression in Tau.P301S mouse model of Alzheimer’s disease and frontotemporal dementia

**DOI:** 10.1186/s13195-018-0378-7

**Published:** 2018-06-19

**Authors:** Mei Ji, Xi-xiu Xie, Dong-qun Liu, Xiao-lin Yu, Yue Zhang, Ling-Xiao Zhang, Shao-wei Wang, Ya-ru Huang, Rui-tian Liu

**Affiliations:** 1 National Key Laboratory of Biochemical Engineering, Institute of Process Engineering, Chinese Academy of Sciences, Haidian District, Beijing, 100190 China; 2 University of Chinese Academy of Sciences, Beijing, 100049 China; 3 Shandong Provincial Key Laboratory of Animal Biotechnology and Disease Control and Prevention, Shandong Agricultural University, Taian, 271018 China

**Keywords:** Alzheimer’s disease, Frontotemporal dementia, Hepatitis B core protein, Truncated tau, Neurofibrillary tangles, Virus-like particles (VLPs), Vaccine

## Abstract

**Background:**

Truncated mis-disordered tau protein plays an important role in the pathogenesis of Alzheimer’s disease (AD) and frontotemporal dementia (FTD). Tau_294–305_, an epitope in the truncated tau, is essential for pathological tau-tau interaction and aggregation. A tau_294–305_-targeted approach may have beneficial effects in the treatment of AD and FTD.

**Methods:**

In this study, we genetically fused tau_294–305_ epitope to the hepatitis B virus core protein (HBc) major immunodominant region (MIR) (with the resultant protein termed T294-HBc), and we subcutaneously immunized a Tau.P301S transgenic mouse model of FTD and AD with T294-HBc four times. The levels and characteristics of antibodies induced by T294-HBc were determined by enzyme-linked immunosorbent assay. The effect of T294-HBc on the cognitive deficits of Tau.P301S mice was tested using the Morris water maze test, novel object recognition, and a Y-maze test. Western blot analysis and IHC were applied to measure the effect of T294-HBc on tau pathologies and neuroinflammation in the mouse brains.

**Results:**

The results showed that T294-HBc self-assembled into HBc chimeric virus-like particles (VLPs) with tau_294–305_ displayed on the surface and that it induced high antibody titers specifically against the mis-disordered truncated tau. Further investigation showed that these antibodies simultaneously bound to microtubule-binding regions 1–4 (MTBR1–4) [tau_263–274,_ tau_294–305_, tau_325–336_, tau_357–368_ and tau_294–305_(P301S)]. Moreover, T294-HBc VLP vaccination significantly ameliorated memory and cognitive decline; reduced the levels of AT8-positive tau, truncated tau monomer, and oligomer; attenuated microgliosis and astrogliosis; and rescued synaptic deficits in Tau.P301S transgenic mice.

**Conclusions:**

T294-HBc VLP vaccine elicited strong immune response and alleviated cognitive deficits and neuropathology progression in Tau.P301S mice, indicating that the T294-HBc VLP vaccine has promising therapeutic potential for the treatment of AD and FTD.

## Background

Alzheimer’s disease (AD) is an age-related neurodegenerative disorder characterized by progressive memory loss [1], intracellular neurofibrillary tangles (NFTs) composed of microtubule-associated protein tau, and extracellular amyloid plaques formed by amyloid-β (Aβ) aggregates in the brain [2]. Unlike Aβ plaques, the amount and extent of NFT pathology positively correlate with the severity of the cognitive deficit of AD [3, 4]. Tau inclusions are also found in other tauopathies that lack Aβ pathology, such as corticobasal degeneration, Pick’s disease, and progressive supranuclear palsy [5]. Notably, mutations in the tau gene (such as *P301S, P301L*) cause some forms of frontotemporal dementia (FTD), indicating that tau dysfunction alone is sufficient to cause neurodegeneration [6]. Current pharmacological treatment of AD is based on cholinesterase inhibitors and memantine. However, this treatment could not halt the disease’s progress [7].

Previous reports suggest that cerebrospinal fluid tau in patients with AD and patients with mild cognitive impairment comprises primarily truncated forms of tau (151–391/2N4R, sequence corresponding to full-length 2N4R tau), which are conformationally different from normal healthy tau and essential for pathologic tau-tau interaction [8–10]. During the development of AD, the mis-disordered truncated tau aggregates to tau tangles and oligomers and drives AD-like neurofibrillary degeneration accompanied by microglial and astroglial activation in the brain [8, 9]. Therefore, therapy targeting mis-disordered truncated tau may be an effective treatment strategy [10]. In the past few years, the field of anti-tau immunotherapies has been galvanized by hundreds of studies trying to optimize the approach for treating AD. Tau-targeted immunotherapies have shown potential for AD treatment, some of which have paved the way to clinical trials [11–14]. Compared with passive immunotherapy, active immunotherapy can provide persistent effects because it uses the immune system to produce long-lasting antibody. Because active immunization with full-length Aβ induced meningitis in clinical trials by a cell-mediated type 1 helper T cell (Th1) immune response [15, 16], the second generation of AD vaccines was developed by conjugating a B-cell epitope of tau or Aβ with a carrier [17, 18]. Previous studies demonstrated that tau_294–305_ is a structural determinant of the truncated tau protein for the pathological tau-tau interaction, and it contains a motif, “HXPGGG,” which localizes not only in tau_299–304_ (within microtubule-binding region 2 [MTBR2]) but also in tau_268–273_ (within MTBR1), tau_330–335_ (within MTBR3), and tau_362–367_ (within MTBR4). A tau_294–305_-targeting approach reduced tau pathology and associated behavioral deficits in transgenic tau rats [17].

Virus-like particles (VLPs) are multimeric nanoparticles that are assembled from viral structural proteins and are biosafe because they lack a viral genome [19]. VLPs contain repetitive high-density viral surface proteins and are an excellent platform from which to present heterologous antigens. Tau_294–305_ is a short peptide with low immunogenicity. To develop a vaccine with tau_294–305_ as an immunogen to induce high antibody titers, tau_294–305_ should be combined with a carrier such as keyhole limpet hemocyanin. Hepatitis B virus core protein (HBc) VLPs are promising carriers because of their high capacity for foreign insertions, high-level generation, and efficient self-assembly in virtually all known homologous and heterologous expression systems, including bacteria and yeast [20–22]. The major immunodominant region (MIR) in HBc is generally used for the insertion of foreign B-cell epitopes to maximally expose these epitopes on the VLP surface and consequently provide the most efficient immunogenic activity. In this study, we fused tau_294–305_ to HBc MIR and assessed its effect on tauopathies in Tau.P301S transgenic mice.

## Methods

### Plasmid construction and protein expression

**Fig. 1:**
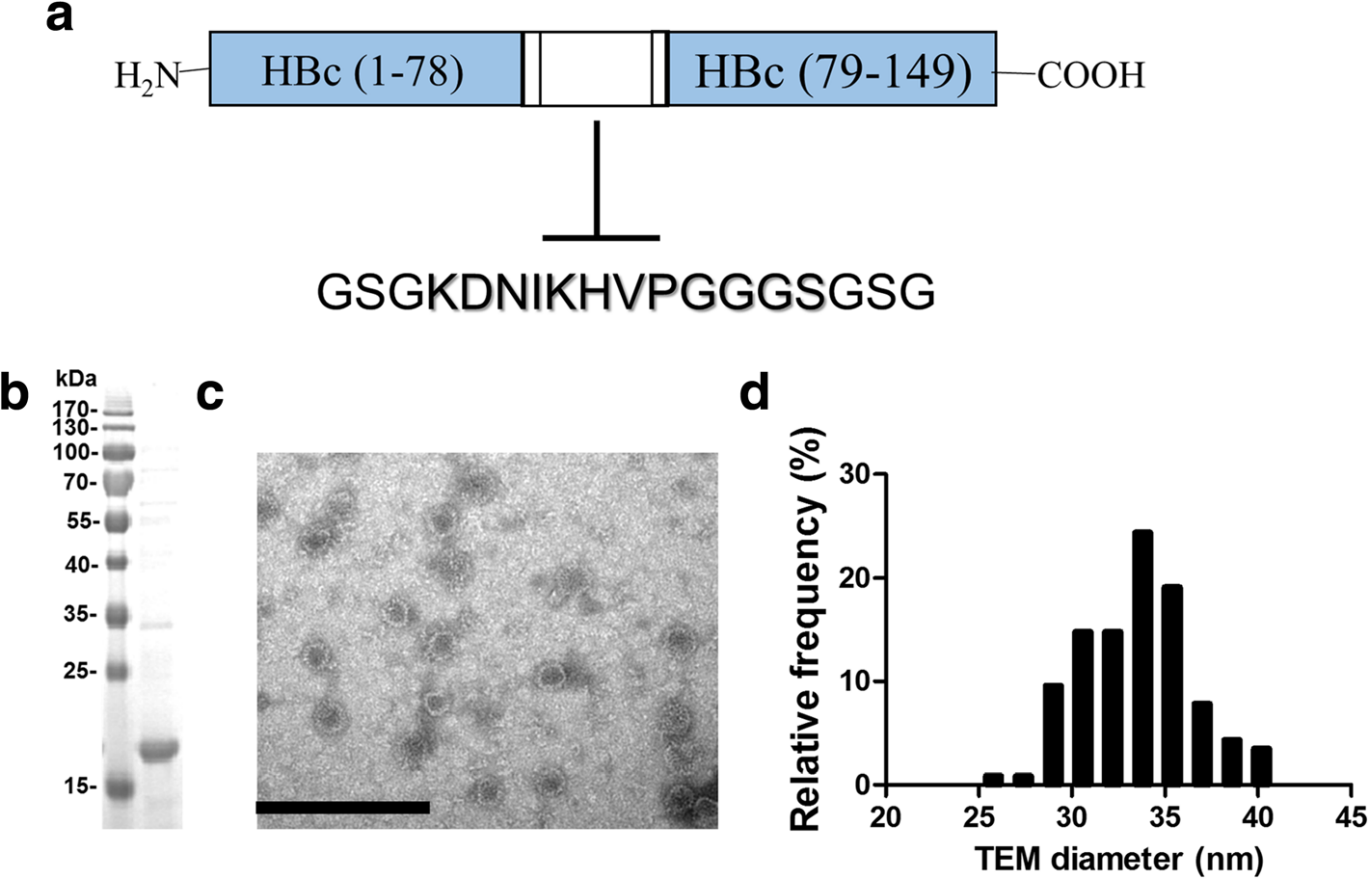
Characterization of the tau_294–305_ epitope to hepatitis B core immunodominant region (T294-HBc) virus-like particle (VLP) vaccine. **a** Schematic representation of the genetic fusion constructs of T294-HBc. The tau_294–305_ epitope was inserted in the major immunodominant region (MIR) between amino acids 78 and 79 with a GSG linker on each side of the epitope. **b** After preparation, T294-HBc VLPs were subjected to 15% SDS-PAGE to detect the purity of T294-HBc. **c** The samples were then applied to copper grids, negatively stained, and imaged by TEM (Hitachi, Tokyo, Japan) at 80 kV and × 60,000 magnification. **d** The size distribution of VLPs determined by TEM was measured using Nano Measurer 1.2 (*n* = 100). Scale bar is 200 nm

A truncated *HBc* gene (coding for amino acids 1 to 149) was cloned into pBR327 vector, which was a kind gift from Professor Andris Kazaks (Latvian Biomedical Research and Study Center, Latvia) [23]. The *tau*_*294–305*_ gene was inserted into the site between the codons for Asp78 and Pro79 in the immunodominant loop region (Fig. 1a). The resultant vector T294-HBc-pBR and HBc-pBR was transformed into *Escherichia coli* BL21 (DE3)-competent cells (Takara, Dalian, China). Cells were cultivated in M9 salt medium supplemented with 1% casamino acids (BD Biosciences, San Jose, CA, USA), 0.2% glucose (Amersco, Solon, OH, USA), 50 μg/ml ampicillin without additional induction of Ptrp for 20–24 hours at 37 °C [24].

For the purification of the VLPs, 2 g of wet cells were resuspended in 40 ml of lysis buffer [50 mM Tris-HCl, 150 mM NaCl, pH 8.0, 5 mM ethylenediaminetetraacetic acid (EDTA), 100 μg/ml phenylmethylsulfonyl fluoride] and then ultrasonicated for 30 minutes at 50% power at 3-second intervals in an ice bath. After centrifugation at 12,000 rpm for 15 minutes, ammonium sulfate was added to the supernatant until 33% saturation was reached. The sample prepared by ammonium sulfate precipitation was subjected to sucrose discontinuous gradient centrifugation at 112,000 × *g* for 16 hours at 4 °C. The collected sucrose solution was then loaded onto a hydroxyapatite column (Bio-Rad Laboratories, Hercules, CA, USA), and the flow-through was collected [25]. Purified protein was concentrated and determined by using a bicinchoninic acid protein assay kit (Pierce, Rockford, IL, USA). The purity of the recombinant protein was analyzed by 15% SDS-PAGE. Full-length tau isoform 2N4R construct was gifted by Professor Virginia M.-Y. Lee [26]. Mis-disordered tau (151–391/2N4R) and full-length tau isoform 2N4R were prepared with *E. coli* BL21 (DE3) as described previously, with some modifications [26, 27].

### TEM assay of VLPs

VLPs (10 μl; 0.2 mg/ml) were applied to 200 mesh copper grids for 5 minutes, blotted with filter paper, and negatively stained with 2% uranyl acetate for 1 minute, then blotted and air-dried. VLPs were imaged using a TEM system (Hitachi, Tokyo, Japan) at 80 kV and 60,000 × magnification. Particle diameter was measured with Nano Measurer 1.2 (*n* = 100).

### Mouse immunization

**Fig. 2:**
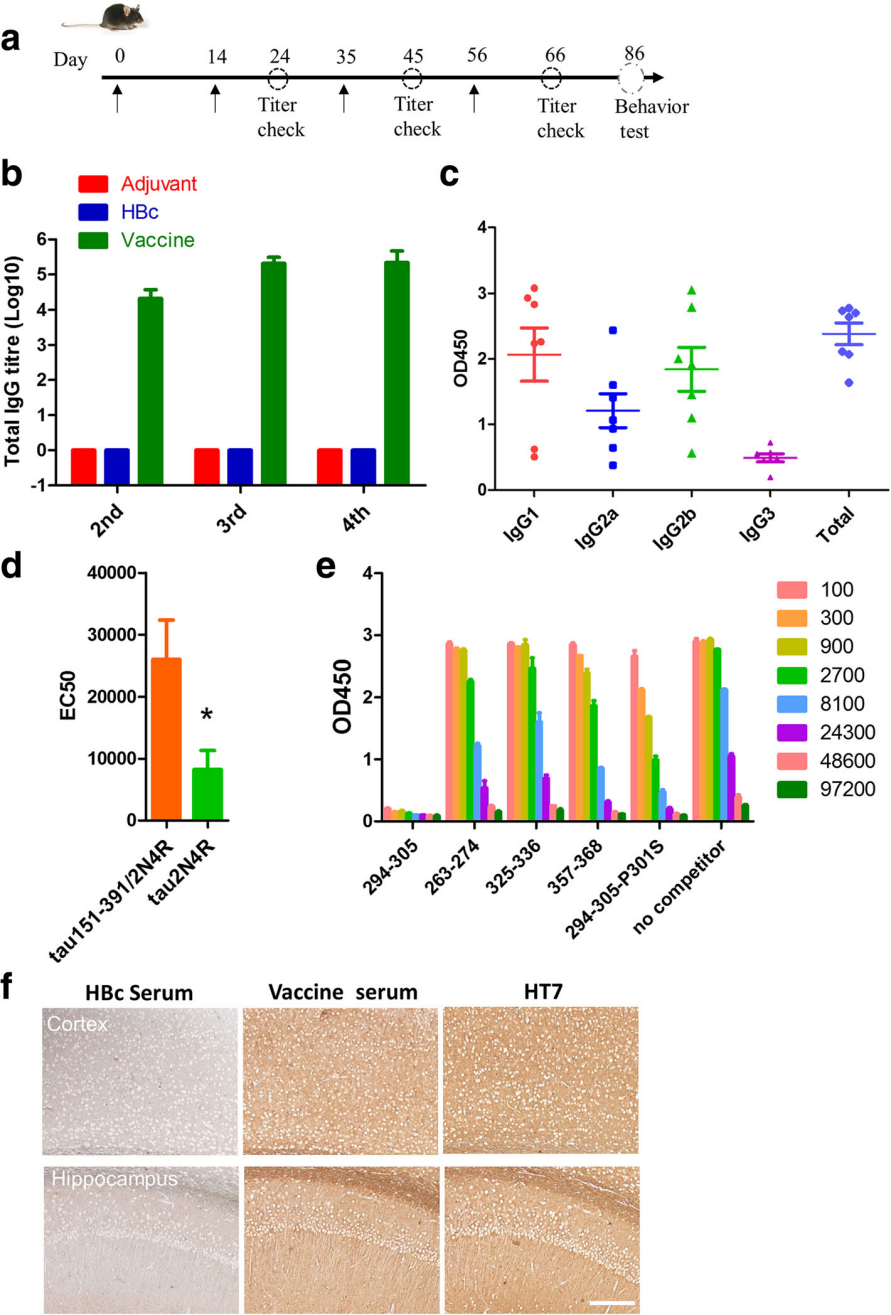
Humoral immune responses induced by tau_294–305_ epitope to hepatitis B core immunodominant region (T294-HBc) virus-like particle (VLP) vaccine. **a** Schematic diagram of the time points for treating Tau.P301S mice (5 months old) (*n* = 7). **b** The antibody titer induced by T294-HBc VLPs. Sera from immunized mice were serially diluted from 1:100 to 1:819,200 in twofold dilution steps and tested in duplicates by enzyme-linked immunosorbent assay (ELISA) against mis-disordered tau (151–391/2N4R). **c** The isotypes of vaccine-induced antibodies. The diluted sera were added to mis-disordered tau (151-391/2N4R)-coated plate and followed by adding HRP–conjugated secondary antibodies. **d** Different binding of serum antibodies to mis-disordered tau (151-391/2N4R) and full-length tau 2N4R. Sera from immunized mice were serially diluted from 1:100 to 1:3906250 in fivefold dilution steps and tested in duplicates by ELISA against mis-disordered tau (151-391/2N4R) and full length tau 2N4R, respectively. EC50, Half-maximal effective concentration. (Statistics were analyzed by student’s t-test, *P<0.05). **e** The binding activity of the antibody elicited by T294-HBc to different tau fragments. The mixture of 200 μM of different tau fragments with diluted sera was added to the mis-disordered tau-coated plate, and then HRP-conjugated goat anti-mouse immunoglobulin G was added. **f** The binding activity of the antibodies elicited by T294-HBc to the brain tissues of Tau.P301S mice. Brain tissue was detected via IHC using serum from HBc (1:200)- or T294-HBc (1:200)-treated mice. Scale bar is 200 μm

Animals were generated by breeding Tau.P301S(1N4R) transgenic male mice with wild-type (WT) female mice under the original C57BL/6×C3H background. All experimental protocols were approved by the institutional animal care and use committee of Tsinghua University. All experiments were performed in accordance with the China Public Health Service Guide for the Care and Use of Laboratory Animals. Offspring were genotyped by polymerase chain reaction of tail DNA. Tau.P301S transgenic mice (5 months old) were randomly assigned to treatment with adjuvant (*n* = 7), HBc (*n* = 7), or T294-HBc (*n* = 7), and their WT littermates (n = 7) were used as a positive control in the behavior test. Mice were inoculated subcutaneously four times at 2-week or 3-week intervals (Fig. 2a). The vaccine and HBc group consisted of 25 μl of Alum Adjuvant (Thermo Fisher Scientific, Waltham, MA, USA) and 75 μl of T294-HBc or HBc (1.33 mg/ml). The adjuvant group was immunized with 25 μl of Alum diluted in 75 μl of PBS. Serum samples were collected before each inoculation and 10 days after the final boost. The effects of T294-HBc on the behavioral and cognitive abilities of Tau.P301S mice were tested 20 days after the last administration.

### Indirect enzyme-linked immunosorbent assay

Serum antibody specific for mis-disordered truncated tau (151–391/2N4R) was detected by enzyme-linked immunosorbent assay (ELISA). Ninety-six-well plates (Dynex Technologies, Chantilly, VA, USA) were coated with 250 ng of recombinant full-length tau isoform 2N4R or pathological truncated tau (151–391/2N4R) per well at 4 °C overnight, then washed twice with PBS and blocked with 3% bovine serum albumin (in 0.05%PBS with Tween-20) for 2 hours at 37 °C. After blocking, the plates were incubated with serial dilutions of the serum (100 μl/well in twofold or fivefold dilution steps) for 1 hour at 37 °C. The bound serum antibodies were detected with horseradish peroxidase (HRP)-conjugated goat antimouse immunoglobulin G (IgG) (Zhongshan Golden Bridge Biotechnology Co., Beijing, China) and chromogenic substrate 3,3′,5,5′-tetramethylbenzidine (Thermo Fisher Scientific).

To determine the isotypes of the specific antibodies produced in response to vaccine, mis-disordered truncated tau (151–391/2N4R) was coated onto ELISA plates, and sera from immunized mice were diluted at 1:6000 and added to the plate, followed by the addition of HRP-conjugated IgG1, IgG2a, IgG2b, and IgG3 (Abcam, Cambridge, UK).

### Competitive ELISA

Ninety-six-well plates (Dynex Technologies) were coated with 250 ng/well of mis-disordered tau (151–391/2N4R). Peptide competitors (> 95% purity; GL Biochem [Shanghai] Ltd., Shanghai, China) were dissolved in PBS to a final concentration of 5 mM. The serum was diluted from 1:100 to 1:97,200 in threefold or twofold dilution in PBS, and 60 μl of the diluted serum solution was mixed with 40 μl of peptide solution (200 μM) in 1.5-ml Eppendorf tubes (Eppendorf, Hamburg, Germany). Antibody/peptide mixtures (100 μl) were then transferred onto ELISA plates and incubated for 1 hour at 37 °C. Bound serum antibodies were detected with HRP-conjugated goat antimouse IgG (Zhongshan Golden Bridge Biotechnology Co.).

### Forced Y-maze test

Forced Y-maze test was conducted using a symmetrical Y-maze made of gray wood, covered with black paper, and consisting of three arms with an angle of 120 degrees. Each arm was marked at the end with a different black-and-white pattern. The three identical arms were randomly designated as the start arm, in which the mouse started to explore (always open); the novel arm, which was blocked during the first trial but open during the second trial; and the other arm (always open). The Y-maze test consisted of two trials separated by an intertrial interval (ITI) to assess spatial recognition memory. The first trial (training) had a 5-minute duration and allowed the mouse to explore only two arms (start arm and other arm) of the maze, with the third arm (novel arm) being blocked. After a 30-minute ITI, the mice were placed back in the maze in the starting arm, with free access to all three arms for 5 minutes for the second trial. All trials were recorded on a videocassette recorder using a ceiling-mounted charge-coupled device (CCD) camera, and the number of entries and time spent in each arm in the video recordings were analyzed.

### Novel object recognition test

Novel object recognition (NOR) is based on the spontaneous tendency of mice to exhibit more interactions with a novel object rather than a familiar object. In the habituation phase, each mouse was allowed to freely explore the open-field arena (a white box 40 cm wide, 40 cm deep, 40 cm high) in the absence of objects. Then, the mouse was removed from the box and placed in its housing cage. During the familiarization period, each mouse was placed in the white box containing two identical objects for 5 minutes and returned quickly to its housing cage. Recognition memory was tested after 24 hours by exposing the mouse to one familiar object and one novel object. The time spent exploring and sniffing each object was recorded.

### Morris water maze test

On training days 1–5, four groups of mice were habituated to a 1.2-m-diameter circular pool with opaque water maintained at 22 ± 1 °C. The mice were allowed to find a platform hidden below the water surface by swimming for 60 seconds for two trials per day. If they didn't  found the platform, the mice were guided to the platform.  All the mouse were allowed to stay on the platform for 10 seconds. The swimming activity of each mouse was automatically recorded via a video tracking system using a video camera (Sony Corp., Tokyo, Japan) mounted overhead. At 24 hours after the last learning trial, the mice were tested for memory retention in a probe trial without the platform.

### Spontaneous Y-maze test

We recorded spontaneous alternation behavior in a Y-maze test to assess short-term memory performance. The maze was same as the forced Y-maze, except that the marker at the end of each arm was changed to eliminate the effects of the former forced Y-maze. This test consisted of a single 5-minute trial in which the mouse was allowed to move freely to all three arms of the Y-maze. The series of arm entries, including possible returns into the same arm, was recorded with a CCD camera connected to a computer. An alternation was defined as entry into all three arms on consecutive occasions. The number of maximum alternations was therefore the total number of arm entries minus 2, and the percentage of alternations was calculated as (actual alternations/maximum alternations) × 100% as described previously [28].

### IHC

Mice were deeply anesthetized with sodium pentobarbital and killed after cardiac perfusion with ice-cold PBS containing heparin (10 U/ml) at the end of the behavior test. The left half of the brain was fixed in 4% paraformaldehyde and embedded in paraffin. Sagittal serial sections of 5-μm thickness were cut on a Leica CM1850 microtome (Leica Biosystems, Buffalo Grove, IL, USA). The sections were dewaxed with antigen retrieval and then treated briefly with 80% (vol/vol) methanol containing 0.3% H_2_O_2_ to prevent endogenous peroxidation. The sections were then blocked with 10% normal goat serum and 0.3% Triton X-100 in PBS to prevent nonspecific protein binding and penetrate cell membranes. Subsequently, the sections incubated with primary antibodies AT8 (1:500; Thermo Fisher Scientific), anti-ionized calcium-binding adaptor molecule-1 (anti-Iba-1) (1:100; GeneTex, Irvine, CA, USA), anti-glial fibrillary acidic protein (anti-GFAP) (1:100; Cell Signaling Technology, Danvers, MA, USA), anti-synaptophysin (1:100; Abcam), sera from vaccine- and HBc-treated mice (1:200) at 37 °C for 1 hour, respectively, followed by incubation with an HRP-labeled secondary antibody at 37 °C for 1 hour. The targets were visualized with 3′-diaminobenzidine substrate. Alexa Fluor 488-labeled secondary antibody (Thermo Fisher Scientific) was used for the detection of synaptophysin levels in the brain sections. All images were collected using a BX60 microscope (Olympus Corp., Shinjuku, Tokyo) with 4 × and 10 × lens objectives. The right half was stored at − 80 °C for Western blot analysis.

### Western blot analysis

The right half brain tissues were dounce-homogenized in radioimmunoprecipitation assay (RIPA) buffer (containing protease inhibitor mixture, 50 mM Tris, pH 7.2, 150 mM NaCl, 5 mM EDTA, and 0.1% SDS) and then centrifuged at 12,000 × *g* for 1 hour at 4 °C. The supernatant (i.e., RIPA-soluble fraction) containing soluble tau was collected. The RIPA-insoluble pellets were washed with 1 M sucrose in RIPA buffer to remove myelin and associated lipids by centrifugation at 100,000 × *g* for 30 minutes at 4 °C. The RIPA-insoluble pellets were then extracted with 2% SDS buffer (50 mM Tris, pH 7.6) [29]. Soluble and insoluble fractions were separated by SDS-PAGE and then transferred onto nitrocellulose membranes. After blocking for 2 hours at room temperature with 5% nonfat milk, the membranes were probed with AT8 (1:1000), tau5 (1:2000), HT7 (1:3000), anti-Iba-1 (1:1000), anti-GFAP (1:1000), anti-synaptophysin (1:1000), anti-GAPDH (1:1000; Cell Signaling Technology), and anti-β-actin (1:1000; MBL, Nagoya, Japan) at room temperature for 1 hour, respectively, and detected with HRP-conjugated anti-mouse or anti-human IgG (1:10,000). The blots were developed using electrochemiluminescence according to the manufacturer’s instructions (Thermo Fisher Scientific), and the optical densities of the bands were determined using IPwin5 Image-Pro Plus software (Media Cybernetics, Rockville, MD, USA).

### Statistical analysis

The data were expressed as mean ± SEM. The time per experiment and samples per group depended on the experiments. We performed ELISAs at least three times, but seven mice per group were analyzed for behavior tests, IHC, and Western blot analysis. To compare the adjuvant group and the vaccine group, one-way analysis of variance (ANOVA) with LSD, two-way ANOVA, Mann-Whitney *U* test, or Student’s *t* test was applied using Prism 5.0 (GraphPad Software, La Jolla, CA, USA) or SPSS 16.0 software (SPSS, Chicago, IL, USA). *P* < 0.05 was considered significant.

## Results

### The preparation of T294-HBc

To improve the immunogenicity of tau_294–305_, we fused it to the HBc MIR region. The epitope was inserted between amino acids 78 and 79 with a GSG linker at each side (Fig. 1a). After three purification steps, SDS-PAGE analysis showed that the purity of T294-HBc was > 95% (Fig. 1b). T294-HBc was then subjected to negative staining for TEM to confirm VLP particle formation. The results showed that the recombinant T294-HBc automatically assembled into VLPs with a diameter of 33.55 ± 2.79 nm (Fig. 1c and d).

### T294-HBc VLPs effectively elicit high-titer antibodies against mis-disordered tau (151–391/2N4R)

Tau.P301S transgenic mice were immunized subcutaneously with 100 μg of T294-HBc VLPs for four times, and the serum antibody titer was detected by ELISA (Fig. 2a). The results demonstrated that the T294-HBc vaccine induced a robust antibody response in mice. In contrast, the adjuvant or HBc alone did not elicit specific antibodies against mis-disordered tau 151–391/2N4R (Fig. 2b). The titers increased with the immunization times and reached a plateau at the third inoculation (Fig. 2b). The geometric mean titer (GMT) of antibodies specific to mis-disordered tau 151–391/2N4R reached high values at 178,700. On the contrary, the EC50 of antibodies recognizing full-length tau 2N4R was much lower than tau 151–391/2N4R, suggesting that the induced antibodies exhibited significantly high binding activity to the mis-disordered tau 151–391/2N4R relative to physiological tau 2N4R (*P* < 0.05) (Fig. 2d).

The isotypes of the antibodies in response to T294-HBc vaccine were detected by ELISA. The results indicated that vaccination of mice with T294-HBc preferentially induced the generation of IgG1 antibody isotypes (Fig. 2c), suggesting that predominant Th2 immune response was involved in T294-HBc immunization.

To detect the ability of serum antibodies to bind to different fragments of tau protein, a competitive ELISA was carried out by mixing 200 μM tau_263–274_ (MTBR1), tau_294–305_ (MTBR2), tau_325–336_ (MTBR3), tau_357–368_ (MTBR4), and tau_294–305_ (P301S) with serum antibodies at different dilutions. As shown in Fig. 2e, besides tau_294–305_, the antibodies elicited by T294-HBc markedly bound to tau_357–368_ and tau_294–305_ (P301S). Moreover, the antibodies also recognized tau_263–274_ and tau_325–336_, but with lower affinity than tau_294–305_, suggesting the therapeutic potential of T294-HBc vaccine in the treatment of AD and FTD.

To further characterize the immune response, we performed IHC using antibody HT7 and the sera from vaccine- and HBc-treated mice. The results showed that HT7 and the sera induced by vaccine bound to the brain tissues of Tau.P301S mice, whereas no positive signal was observed with the addition of the sera from HBc-treated mice, indicating that antibodies induced by T294-HBc vaccine recognized tau pathology (Fig. 2f).

### T294-HBc VLP immunization improved cognitive capacity in Tau.P301S mice

**Fig. 3:**
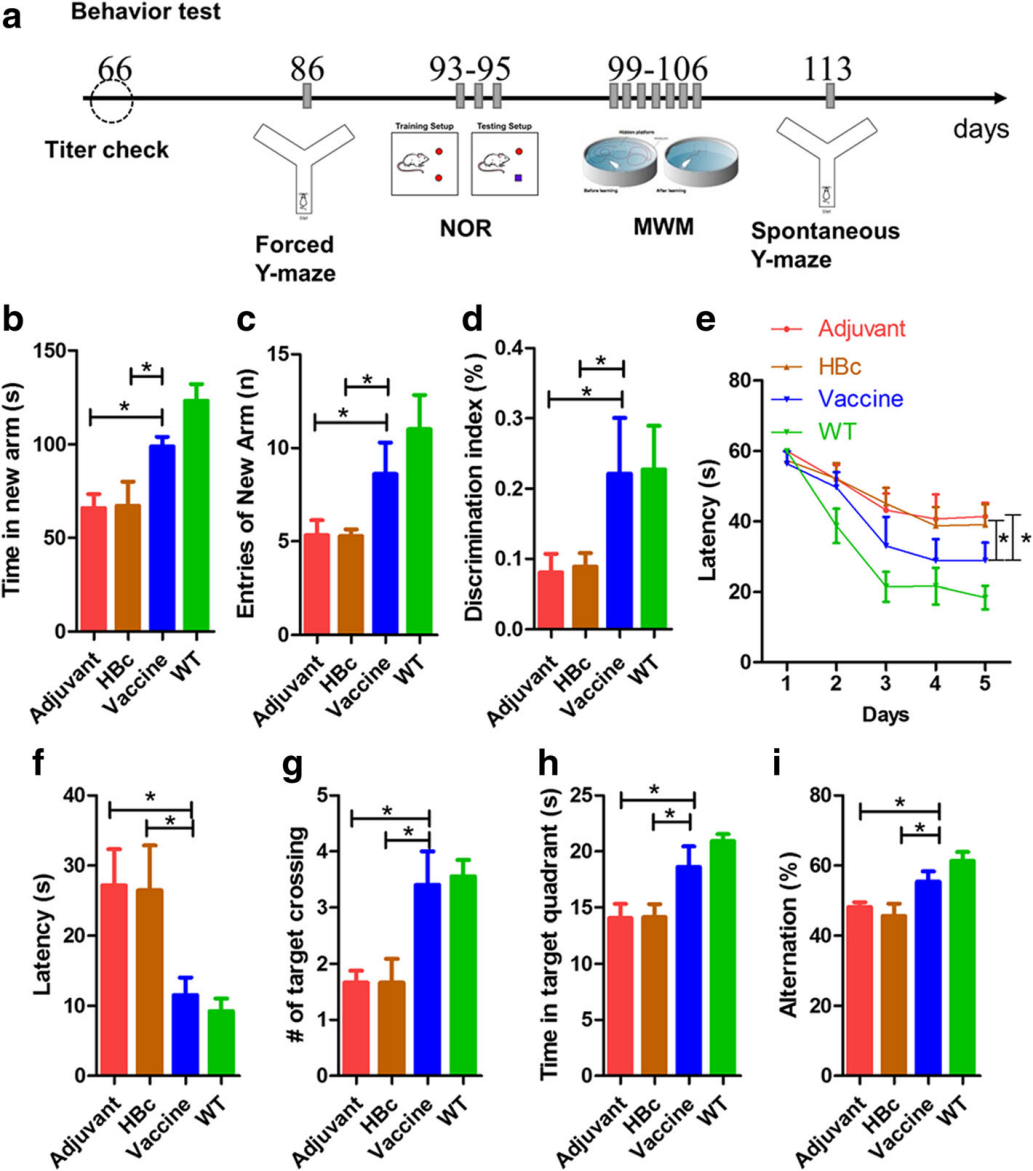
Tau_294–305_ epitope to hepatitis B core immunodominant region (T294-HBc) vaccination improved cognitive capacity in Tau.P301S mice. **a** Schematic diagram of behavioral test battery. **b** In the forced Y-maze, the time that mice spent in the new arm was recorded in 30-minute intertrial intervals. One-way analysis of variance (ANOVA) with LSD was used for statistical analysis. c The number of mice entries to the new arm. Statistics were analyzed by Mann-Whitney *U* test. **d** The learning and memory of Tau.P301S mice tested by novel object recognition (NOR). NOR discrimination index was determined by calculating (Time_novel_ − Time_old_)/(Time_novel_ + Time_old_), where 0 equals no preference for either object. One-way ANOVA with LSD was used for statistical analysis. **e** Latency to reach the hidden platform in the acquisition period of the Morris water maze (MWM). For 5 consecutive days, each mouse was subjected to two 60-second trials per day to find the hidden platform. The recorded data were analyzed and showed the changes of escape latency to find the hidden platform over the 5 days of training. Statistical significance was analyzed by two-way ANOVA. **f** Latency to reach the hidden platform location in the probe trial of the MWM without the platform. One-way ANOVA with LSD was used for statistical analysis. **g** The number of times of crossing the platform location during the memory trial in the MWM probe test without the platform. Statistics were analyzed by Mann-Whitney *U* test. **h** The time spent in the target quadrant in the MWM probe test. One-way ANOVA with LSD was used for statistical analysis. **i** The short-term memory of Tau.P301S mice. Spontaneous Y-maze tests were conducted to assess short-term memory of Tau.P301S mice treated with or without T294-HBc virus-like particles. The percentage of alternation was calculated as (actual alternations/maximum alternations) × 100%. One-way ANOVA with LSD (compared with adjuvant- and HBc-treated mice; *P < 0.05) was used for statistical analysis

To evaluate the effect of T294-HBc immunization on Tau.P301S mice, we carried out a behavioral test battery, including a forced Y-maze test, NOR, Morris water maze (MWM), and spontaneous Y-maze test (Fig. 3a) [28]. The forced Y-maze test was performed to examine the effects of vaccine treatment on short-term memory. Compared with the adjuvant- and HBc-treated mice, T294-HBc-immunized mice showed a significant increase (*P* < 0.05) in both time spent and number of entries in the new arm, indicating that T294-HBc improved short-term memory in Tau.P301S mice (Fig. 3b, c).

NOR was performed to further evaluate the effects of the vaccine on mouse memory. Compared with the adjuvant- and HBc-treated mice, the mice treated with T294-HBc VLPs spent more time on the novel object than on the familiar object (*P* < 0.05) (Fig. 3d).

The MWM test was conducted to assess the effects of T294-HBc immunization on the spatial memory and learning ability of Tau.P301S mice. During the acquisition phase of the test, the mice were trained to search for the hidden platform for 5 days. Compared with the adjuvant- and HBc-treated Tau.P301S mice, mice treated with T294-HBc readily found the location of the hidden platform after 2 days of training (*P* < 0.05) (Fig. 3e). After the last training, the platform was removed, and the mice were given 1 minute to find the location of the missing platform for the probe trial. T294-HBc-treated mice exhibited spatially oriented swimming behavior and shorter escape latencies (Fig. 3f) and increased number of target crossings (Fig. 3g), and they spent more time in the target quadrant (Fig. 3h), indicating that T294-HBc substantially improved the spatial memory of Tau.P301S transgenic mice. No significant difference in the swimming speed was observed within mouse groups in the training period and the probe trial session.

Spontaneous alternation using a Y-maze is a further test for habituation and spatial working memory. The percentage of alternation of T294-HBc-treated mice in the spontaneous Y-maze test was significantly increased compared with that of adjuvant- and HBc-treated mice, indicating that short-term memory was rescued in Tau.P301S model mice by T294-HBc VLP immunization (Fig. 3i). Compared with the adjuvant group, the HBc group did not show a significant effect on antibody response and animal behavior, and we did not further detect the pathology in the brains of the HBc group.

### T294-HBc VLP immunization reduced tau aggregates in Tau.P301S mice

**Fig. 4:**
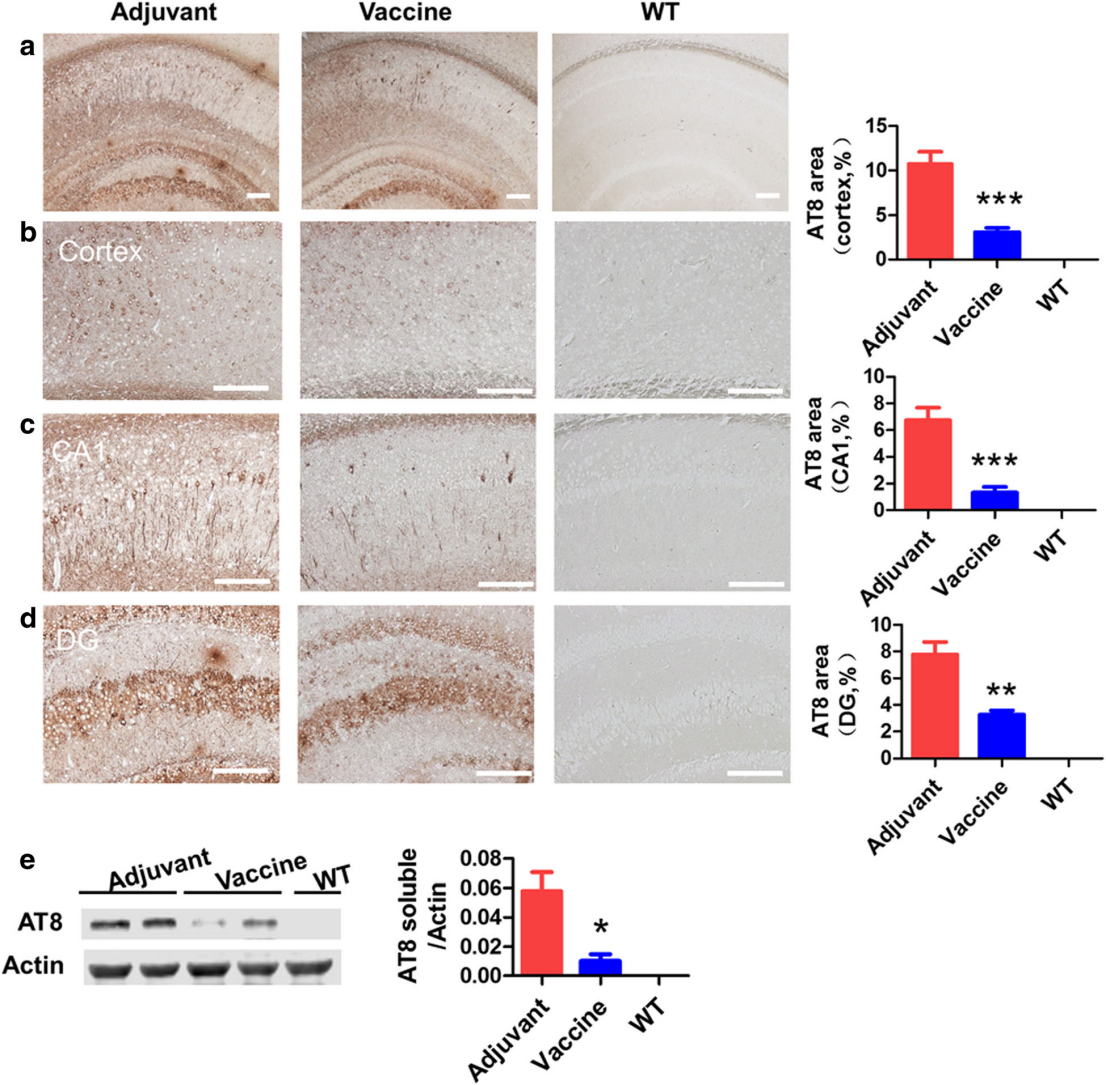
Active immunotherapy with tau_294–305_ epitope to hepatitis B core immunodominant region (T294-HBc) virus-like particle (VLP) vaccine reduced AT8-positive aggregates in Tau.P301S mice. AT8-positive aggregates in brain of mice treated with or without vaccine and of wild-type (WT) mice were detected by using AT8 (1:500) antibody. The AT8-positive aggregates in brain (**a**), cortex (**b**), and hippocampal CA1 (**c**) and dentate gyrus (DG) regions (**d**) were then quantified by morphometric analysis. **e** AT8-positive aggregates levels in soluble brain homogenates. The AT8-positive aggregates levels in the brain homogenates were detected by Western blotting using antibody AT8 (1:3000). The signals of Western blot were quantified using IPwin5 Image-Pro Plus software, and β-actin was used as a control. Scale bar is 200 μm (compared with adjuvant-treated mice; *P < 0.05 **P < 0.01, ***P < 0.001, Student’s *t* test)

We detected AT8-positive aggregates in mouse brains by IHC and Western blotting using anti-phosphorylated tau antibody AT8 to investigate the therapeutic effect of vaccine on the Tau.P301S mice. WT mice did not show any tau pathology in the brain, whereas Tau.P301S mice showed severe AT8-positive aggregates in the cortex and in the hippocampal CA1 and dentate gyrus (DG) regions (Fig. 4). However, T294-HBc VLP vaccine significantly reduced AT8-positive aggregates in these regions. Western blotting also showed that T294-HBc VLPs significantly decreased the levels of AT8-positive aggregates in the brains of mice (Fig. 4e). These results demonstrate that T294-HBc VLPs decreased tau pathology in the mouse brains.

### T294-HBc VLP immunization reduced RIPA-insoluble tau levels in Tau.P301S mice

**Fig. 5:**
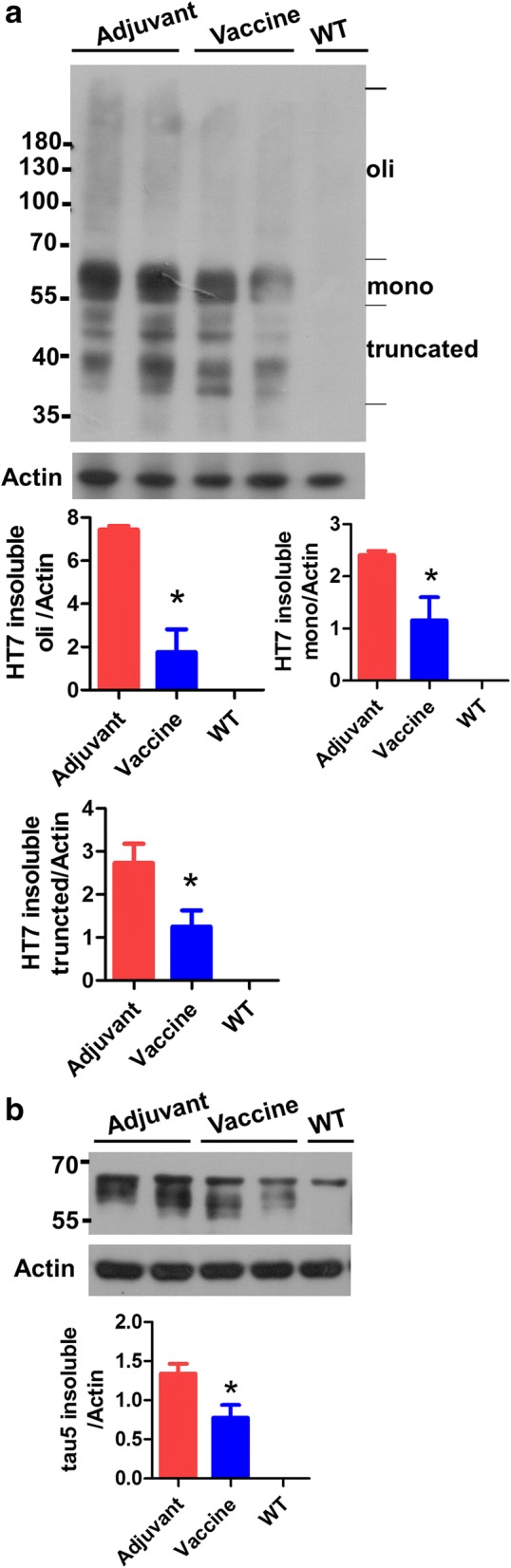
The effect of tau_294–305_ epitope to hepatitis B core immunodominant region (T294-HBc) virus-like particle (VLP) immunization on the levels of insoluble tau in Tau.P301S. The radioimmunoprecipitation assay buffer (RIPA)-insoluble tau in mouse brain homogenates was subjected to SDS-PAGE and then transferred onto nitrocellulose membrane. The tau protein was probed by human tau-specific antibody HT7 (1:3000) (**a**) and total tau antibody tau 5 (1:2000) (**b**), respectively. The Western blot signals were quantified using IPwin5 Image-Pro Plus software, and β-actin was used as a control (compared with adjuvant-treated Tau.P301S mice; *P < 0.05, Student’s *t* test)

To assess the effects of T294-HBc immunization on the levels of different tau species in mouse brains, we detected the RIPA-insoluble tau with human tau-specific antibody HT7 and total tau antibody tau5. Consistent with previous results, T294-HBc significantly decreased the levels of highly phosphorylated forms of truncated tau (30 to 36 kDa), tau oligomers (above 36 kDa), and full-length tau in insoluble tau (Fig. 5a, b). Western blot analysis probed by tau5 antibody showed that WT mice had a band of about 70 kDa corresponding to the molecular weight of mouse tau. Compared with adjuvant-treated mice, T294-HBc-immunized mice had a significant decrease in both human and phosphorylated tau.

### T294-HBc VLP immunization reduced glial cell activation in Tau.P301S mice

**Fig. 6:**
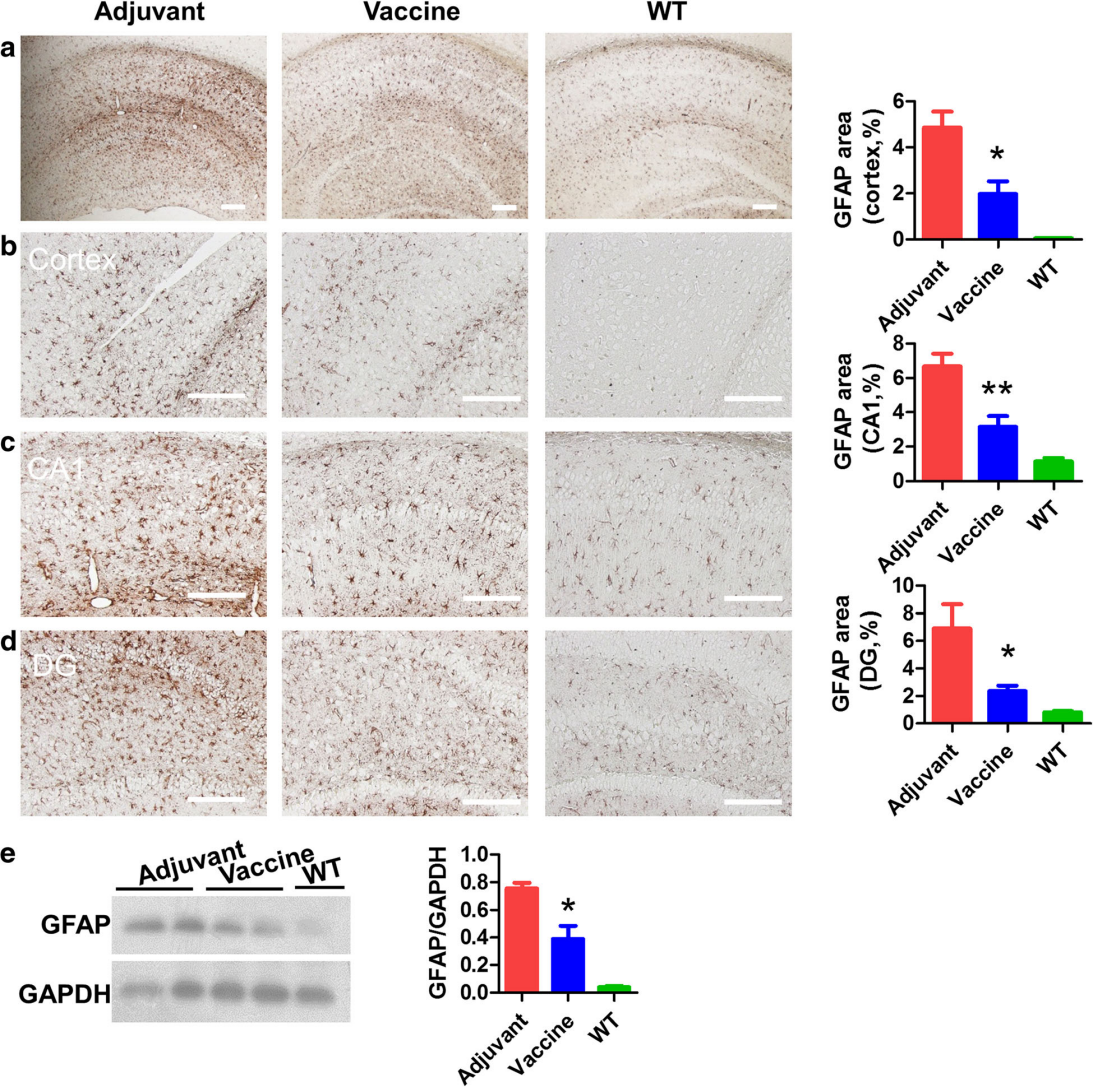
Active immunotherapy using tau_294–305_ epitope to hepatitis B core immunodominant region (T294-HBc) virus-like particle (VLP) vaccine reduced astrogliosis in Tau.P301S mice. Astrogliosis in brain of mice treated with or without vaccine and of wild-type (WT) mice was detected by IHC and Western blotting with anti-glial fibrillary acidic protein (anti-GFAP) antibody. The astrogliosis in brain (**a**), cortex (**b**), and hippocampal CA1 (**c**) and dentate gyrus (DG) regions (**d**) was quantified by morphometric analysis. **e** GFAP levels detected by Western blot analysis. The Western blotting signals were quantified using IPwin5 Image-Pro Plus software, and GAPDH was used as a control. Scale bar is 200 μm (compared with adjuvant-treated mice; *P < 0.05, **P < 0.01, Student’s *t* test)

**Fig. 7:**
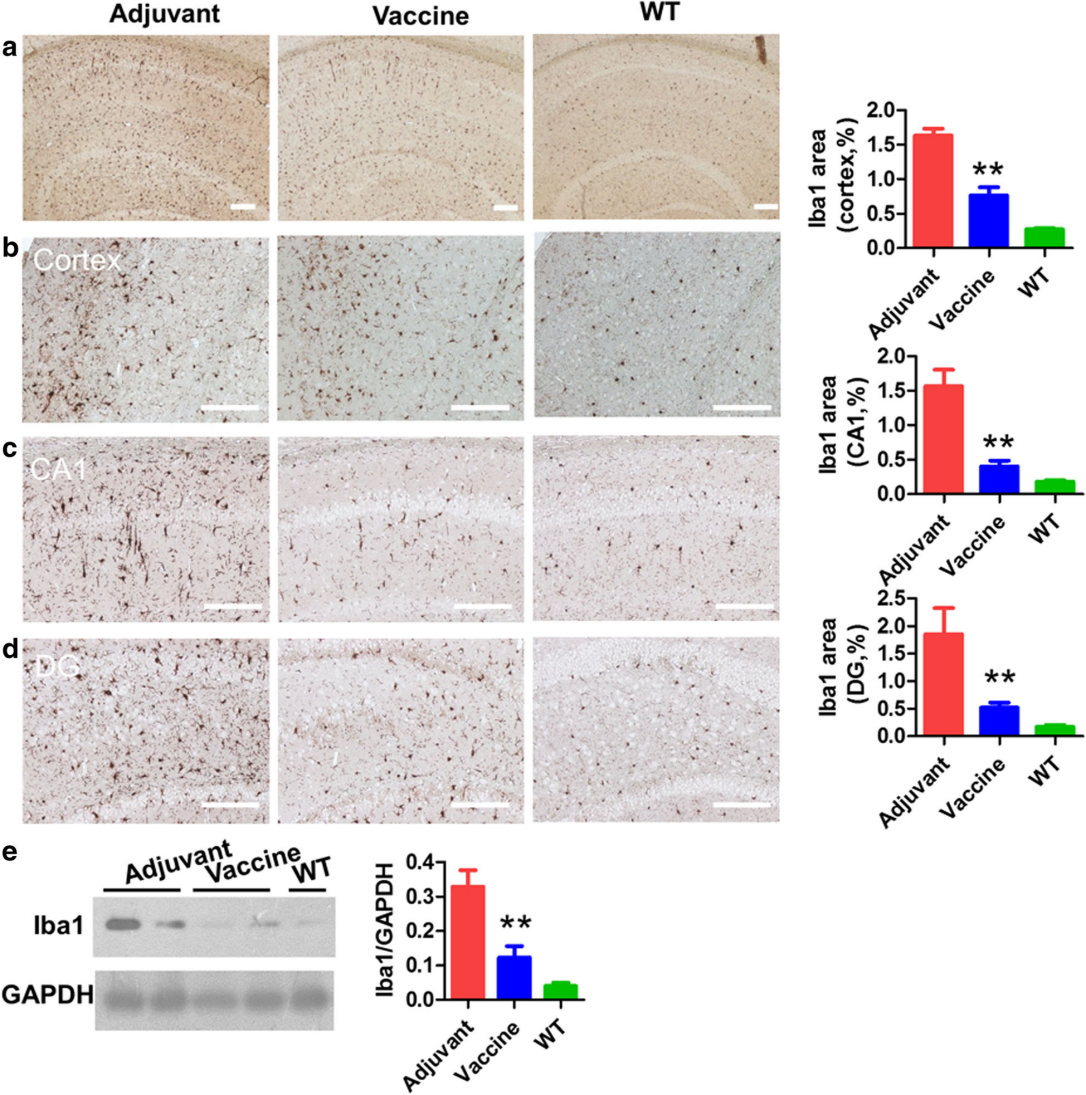
Active immunotherapy by tau_294–305_ epitope to hepatitis B core immunodominant region (T294-HBc) virus-like particle (VLP) vaccine reduced microgliosis in Tau.P301S mice. Microgliosis in brain of mice treated with or without vaccine and of wild-type (WT) mice was detected by IHC and Western blotting with anti-ionized calcium-binding adaptor molecule-1 (anti-Iba-1) antibody. Microgliosis in the brain (**a**), cortex (**b**), and hippocampal CA1 (**c**) and dentate gyrus (DG) regions (**d**) was quantified by morphometric analysis. **e** Iba-1 levels detected by Western blot analysis. The Western blotting signals were quantified using IPwin5 Image-Pro Plus software, and GAPDH was used as a control. Scale bar is 200 μm (compared with adjuvant-treated mice; **P < 0.01, Student’s *t* test)

The infiltration of activated astrocytes and microglia is linked to the pathogenesis of Tau.P301S mice. We stained astrocytes and microglia with antibodies against GFAP and Iba-1, respectively, to address whether vaccine treatment was effective for astrogliosis and microgliosis in the brains of Tau.P301S transgenic mice. The results showed that T294-HBc active immunotherapy reduced the number of activated astrocytes in the cortex and in the hippocampal CA1 and DG regions (Fig. 6). Consistently, our Western blotting results also showed that T294-HBc immunization decreased GFAP protein levels (Fig. 6e). Moreover, T294-HBc reduced the activated microglial levels in the cortex and in the hippocampal CA1 and DG regions (Fig. 7). The results of Western blotting also showed a decrease in microgliosis (Fig. 7e). These results indicated that T294-HBc immunization attenuated neuroinflammation in Tau.P301S mice by targeting pathogenic mis-disordered tau [30].

### Active immunotherapy by T294-HBc VLP vaccine rescued synaptic deficits in Tau.P301S mice

**Fig. 8:**
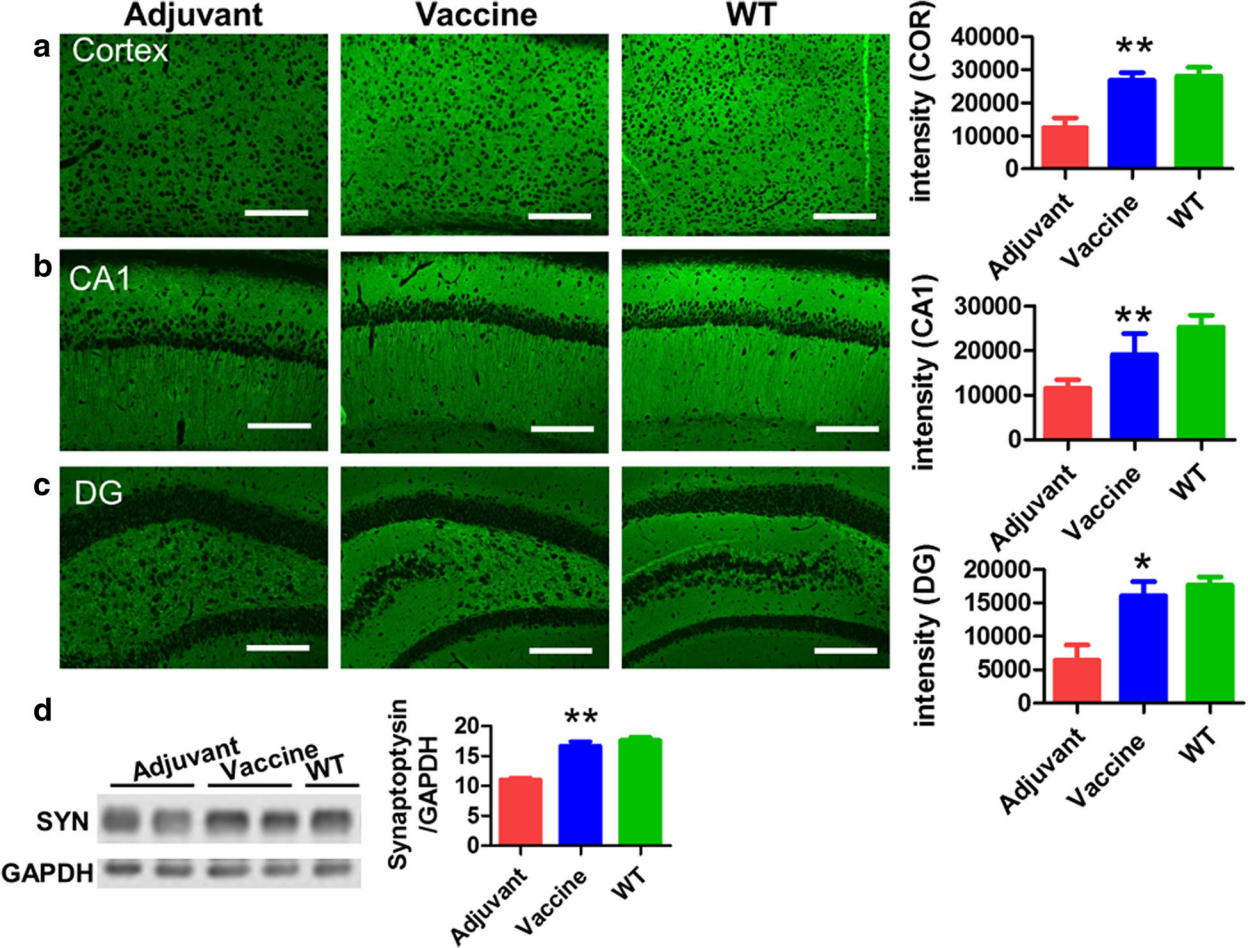
Active immunotherapy using tau_294–305_ epitope to hepatitis B core immunodominant region (T294-HBc) virus-like particle (VLP) vaccine rescued synaptic deficits in Tau.P301S mice. Presynapses in brain of mice treated with or without vaccine and of wild-type (WT) mice were detected by IHC and Western blotting with antisynaptophysin antibody. Synaptophysin in the cortex (**a**) and the hippocampal CA1 (**b**), and dentate gyrus (DG) regions (**c**) was quantified using IPwin5 Image-Pro Plus software, and results are shown in arbitrary units (AU). **d** Synaptophysin levels detected by Western blot analysis. The Western blotting signals were quantified using IPwin5 Image-Pro Plus software, and GAPDH was used as a control. Scale bar is 200 μm (compared with adjuvant-treated mice; *P < 0.05, **P < 0.01, Student’s *t* test)

With the development of tauopathies in Tau.P301S mice, synapses are damaged owing to the toxicity of pathogenic tau [31]. To assess whether the reduction of pathogenic mis-disordered tau can attenuate synaptic deficits, in the present study we used antisynaptophysin antibody to detect the effect of T294-HBc VLP vaccine on the synapses. Significant increases in synaptophysin levels were observed in the cortex and in the hippocampal CA1 and DG regions in T294-HBc-treated mice compared with adjuvant-treated mice (Fig. 8). Western blot analysis also showed an increase in synaptophysin levels in brain homogenates of T294-HBc-treated mice relative to adjuvant-treated mice (Fig. 8d). These results indicate that T294-HBc VLP immunization rescued synaptic deficits in Tau.P301S mice.

## Discussion

Both hyperphosphorylated and truncated tau play a critical role in AD pathogenesis. Authors of many reports selectively targeted phosphorylated tau (phospho-tau) epitopes, including phospho-Ser396/phospho-Ser404 [32–34], phospho-Ser422 [35], or phospho-Thr231/phospho-Ser235 [36]. However, most  phosphoepitopes of tau such as phospho-Ser404 are present in healthy human brains [37], which raises concerns about the safety of immunotherapies targeting those phospho-tau species. Truncated tau is a pathogenic tau present in AD brains but not in normal human brains; thus, targeting truncated tau may be a more promising approach [17]. In this study, we chose tau_294–305_ as our targeting epitope because it is a structural determinant of the truncated tau protein for the pathological tau-tau interaction. This epitope contains a motif, “HXPGGG,” that localizes not only in tau_299–304_ (within MTBR2) but also in tau_268–273_ (within MTBR1), tau_330–335_ (within MTBR3), and tau_362–367_ (within MTBR4) [17, 38]. Therefore, the antibodies induced by tau_294–305_ may simultaneously bind to MTBR1–4.

HBc VLP is a widely used carrier to generate putative vaccines. HBc-based malaria and influenza vaccines have entered into clinical trials, and they were well tolerated. To avoid T-cell autoimmunity likely induced by full-length Aβ, we developed a tau vaccine (T294-HBc VLP vaccine) by genetically fusing a B-cell epitope of tau (tau_294–305_) to HBc MIR. In our tau vaccine, the Th epitopes were derived exclusively from the VLP carrier protein, and the vaccine was formulated with Alum adjuvant that promoted the Th2 immune response. As expected, the antibody induced by T294-HBc VLPs was predominantly the IgG1 isotype, which indicates that the immune response to the vaccine mainly involved the Th2 phenotype and that T294-HBc VLPs may be a safe vaccine type. Moreover, T294-HBc VLPs formed uniform nanoparticles with a diameter of approximately 33.55 nm and elicited robust and specific antibodies against mis-disordered tau in mice.

FTD, the second most common form of dementia before the age of 65, is caused by P301S/L mutated tau. A previous report showed that tau-targeting vaccine AADvac1 improved cognition and reduced tauopathy in a transgenic rat model expressing human truncated tau [17]. Although this transgenic rat model displayed some tau pathology and motor and behavioral deficits, it still could not mimic some incidence and progression of tauopathy in AD and FTD. Moreover, AADvac1 induced antibodies against tau_294–305_, but whether the vaccine has a beneficial effect on an FTD model with P301S/L mutation remains unknown. To explore the effects of T294-HBc VLP vaccine on the animal models of FTD and AD, we applied the vaccine to the Tau.P301S transgenic mouse model, which is widely used to mimic the incidence and progression of FTD and AD and recapitulates the essential molecular and cellular features of the human tauopathies, including truncated tau generation, hyperphosphorylation, tau filament formation, and neurodegeneration [31, 39]. Woerman et al.’s report showed that the PS19 mouse model exhibited great variability in pathology onset at ages over 31 weeks, but it was relatively uniform before age 30 weeks [40]; the mice at 22 weeks of age that we used conformed the latter age range. Our results showed that T294-HBc vaccine improved cognition and memory of Tau.P301S mice and reduced the levels of truncated tau monomer, oligomer, and hyperphosphorylated tau. Our observations are in agreement with previous findings that passive immunization with tau antibodies against pathological tau forms improved cognition of AD mice and reduced the levels of hyperphosphorylated tau [41, 42]. Although the antibodies induced by T294-HBc VLPs recognized tau_294–305_ (P301S) with lower affinity, they could also bind to tau_294–305_, tau_263–274_, tau_325–336_, and tau_357–368_, resulting in beneficial effects on Tau.P301S mice.

The accumulation of NFTs can cause inflammation and synapse loss in Tau.P301S mice [31]. However, the T294-HBc VLP-immunized mice exhibited lower levels of astrogliosis and microgliosis and higher levels of synaptophysin than adjuvant-treated mice, leading to attenuation of cognitive deficits and neuropathology in the transgenic mice. Several mechanisms could explain the antibody-mediated clearance of the mis-disordered tau in vivo [35, 43]. The direct targeting mechanism proposes that a small amount of serum antibodies can cross the blood-brain barrier, bind to the mis-disordered tau, and then induce the phagocytosis of the antigen-antibody complexes via the Fc portion of the antibody [44]. The pathologic tau can propagate from cell to cell and induce the aggregation of pathologic tau in the other neurons [45], and the clearance of mis-disordered tau by our vaccine results in the inhibition of the NFT formation.

## Conclusions

In summary, our study indicates that the HBc VLP-based T294-HBc vaccine exerted favorable effects on cognition and neuropathology in the Tau.P301S transgenic mouse model by inducing high titers of antibodies against truncated tau; decreasing the levels of truncated tau monomer, oligomer, and hyperphosphorylated tau; increasing synaptophysin levels; and suppressing microgliosis and astrogliosis in mouse brains. Moreover, T294-HBc VLP-induced antibodies could simultaneously bind to MTBR 1–4 [tau_263–274_, tau_294–305_, tau_325–336_, tau_357–368_, and tau_294–305_(P301S)], indicating that this vaccine has promising therapeutic potential for the treatment of FTD and AD.

## Abbreviations

AD: Alzheimer’s disease
ANOVA: Analysis of variance
Aβ: β-Amyloid peptide
CCD: Charge-coupled device
DG: Dentate gyrus
EC_50_: Half-maximal effective concentration
ECL: Enhanced chemiluminescence
EDTA: Ethylenediaminetetraacetic acid
ELISA: Enzyme-linked immunosorbent assay
FTD: Frontotemporal dementia
GFAP: Glial fibrillary acidic protein
GMT: Geometric mean titer
HBc: Hepatitis B virus core protein
HRP: Horseradish peroxidase
Iba-1: Ionized calcium-binding adaptor molecule-1
IgG: Immunoglobulin G
ITI: Intertrial interval
MIR: Major immunodominant region
MTBR: Microtubule-binding region
MWM: Morris water maze
NFTs: Neurofibrillary tangles
NOR: Novel object recognition
RIPA: Radioimmunoprecipitation assay
Th: Helper T cell
VLPs: Virus-like particles
WT: Wild type
